# Comparison of Long-Term Outcomes of the Lamellar and Penetrating Keratoplasty Approaches in Patients with Keratoconus

**DOI:** 10.3390/jcm10112421

**Published:** 2021-05-29

**Authors:** Dominika Janiszewska-Bil, Barbara Czarnota-Nowakowska, Katarzyna Krysik, Anita Lyssek-Boroń, Dariusz Dobrowolski, Beniamin Oskar Grabarek, Edward Wylęgała

**Affiliations:** 1 Optegra Eye Clinic, 40-101 Katowice, Poland; 2 Department of Ophthalmology, St. Barbara Hospital, Trauma Centre, 41-200 Sosnowiec, Poland; kkrysik@gmail.com (K.K.); anitaboron3@gmail.com (A.L.-B.); dardobmd@wp.pl (D.D.); 3 Optegra Eye Clinic, 61-101 Poznań, Poland; barbara.czarnota@icloud.com; 4 Department of Ophthalmology, Faculty of Medicine in Zabrze, University of Technology, 41-800 Zabrze, Poland; 5 Chair and Clinical Department of Ophthalmology, Division of Medical Science in Zabrze, Medical University of Silesia in Katowice, 40-760 Katowice, Poland; ewylegala@sum.edu.pl; 6 Department of Ophthalmology, District Railway Hospital, 40-760 Katowice, Poland; 7 Department of Histology, Cytophysiology and Embryology, Faculty of Medicine, University of Technology in Katowice, 41-800 Zabrze, Poland; bgrabarek7@gmail.com

**Keywords:** keratoconus, deep anterior lamellar keratoplasty, penetrative keratoplasty

## Abstract

We compared the visual and refractive outcomes, intraocular pressure (IOP), endothelial cell loss (ECL), and adverse events in keratoconus patients after deep anterior lamellar keratoplasty (DALK) and penetrating keratoplasty (PK) with the best corrected visual acuity (BCVA) below 0.3 (logMAR 0.52). This is a prospective, comparative cohort study of 90 eyes (90 patients) with a clinical diagnosis of keratoconus. Patients underwent a complete eye examination before the surgical approach, 6 and 12 months postoperatively that consisted of BCVA, refractive astigmatism (AS), central corneal thickness (CCT), IOP, and ECL. Secondary outcomes were adverse events related to the surgical procedure. With lower ECL and less adverse events, DALK was revealed to be beneficial over PK with similar visual outcomes. Results: There was no significant difference between the BCVA in the DALK and PK groups (at 6 months: 0.49 ± 0.17 vs. 0.48 ± 0.17; *p* = 0.48; at 12 months: 0.54 ± 0.17 vs. 0.52 ± 0.14; *p* = 0.41). The mean value of AS was significantly lower after the PK procedure when compared to DALK, after both 6 and 12 months of follow up (*p* < 0.001). The CCT in the DALK group was significantly lower when compared to the PK group (at 6 months: 452.1 ± 89.1 µm vs. 528.9 ± 69.9 µm, *p* < 0.0001; at 12 months: 451.6 ± 83.5 µm vs. 525.5 ± 37.1 µm). The endothelial cell loss at 12 months after surgery was significantly lower after DALK when compared to PK (*p* < 0.0001). DALK transplantation should be considered as an alternative procedure in the surgical treatment of keratoconus.

## 1. Introduction

Keratoconus is one of the most frequent, inherited, non-inflammatory, bilateral, and asymmetric corneal ectasias with a typical onset during puberty [1]. Due to its progressive nature, keratoconus is a cause of significant visual impairment in both young males and females, irrespective of race [2]. Corneal transplantation is a method of choice in keratoconus patients with advanced astigmatism that is unable to be corrected with spectacles or contact lenses as well as in patients with pronounced corneal opacities [1,2]. The penetrating keratoplasty (PK) method used to be the only standard surgical procedure for patients with advanced keratoconus.

The development of new keratoplasty techniques (i.e., deep anterior lamellar keratoplasty, DALK) made it possible to minimize the traumatization of the posterior layers of the cornea, which are commonly disease-free in the case of keratoconus [3]. The DALK procedure is one of the modern surgery techniques that allows replacement of the affected anterior corneal layers with a morphologically matching donor tissue and with preservation of the host endothelial layer [3]. Lamellar corneal transplantations not only reduce the invasiveness of the surgery but also, in an essential manner, limit the possibility of donor antigen presentation to the immunocompetent cells present in the anterior chamber [3]. 

In 1999, Anwar & Teichman introduced modification of the DALK technique, described as the big-bubble method [4]. During this surgical procedure, the separation of Descemet’s membrane from the posterior corneal stroma is performed by the injection of air into the deep corneal stroma. Currently, Anwar’s technique remains the most frequently performed method of anterior lamellar keratoplasty [5]. In our presented study, we compare the visual outcomes of two cohort groups of patients who underwent the DALK or PK procedure for advanced keratoconus.

## 2. Materials and Methods

The study was conducted according to the guidelines of the Declaration of Helsinki, and approved by the Institutional of the Bioethical Committee operating at the Regional Medical Chamber in Krakow; no. 68/KBL/OIL/2020 was obtained for this study. Each of patients agreed to participate in the study, and the conscientious consent form was signed by each of the participants. The patients who were unable to take conscientious decisions on their own will were excluded from the study.

### 2.1. Patients

This was a prospective, comparative cohort study of 90 eyes (90 patients) with a clinical diagnosis of keratoconus. Only patients with stages of disease qualifying for keratoplasty were included into the study groups. Corneal transplantations were performed between January 2009 and January 2020 by the same surgeon (WE). The patients were divided into two study groups based on the inclusion criteria presented in Table 1.

DALK group consisted of 50 eyes (50 patients, including 22 females and 28 males) at ages between 19 and 43 years old (mean age 28.6 ± 5.9 years old). These patients underwent the DALK big-bubble technique. The other group of patients (PK group) consisted of 40 eyes (40 patients, including 16 females and 24 males) at ages between 19 and 43 years old (mean age 28.4 ± 8.6 years old). These patients underwent the PK procedure. Patients were examined by the same ophthalmologist (J-BD) at three time-points: preoperatively and 6 and 12 months postoperatively.

### 2.2. Surgical Procedures

All surgical procedures were performed under general and topical anesthesia. In the big-bubble technique, the host cornea was trephined at approximately 80% of the thickness (7.25 mm Hessburg–Barron vacuum trephine, Barron Precision Instruments, Grand Blanc, MI, US). A partial anterior keratectomy was performed using a crescent knife, allowing for the insertion of a 30-gauge needle (bent at its one third distal part) attached to a 2-mL sterile air-filled syringe. The needle was inserted paracentrally into the corneal stroma and directed to the center of the cornea. Sterile air was injected carefully into the deep corneal stroma, followed by paracentesis of the formed bubble. 

**Figure 1:**
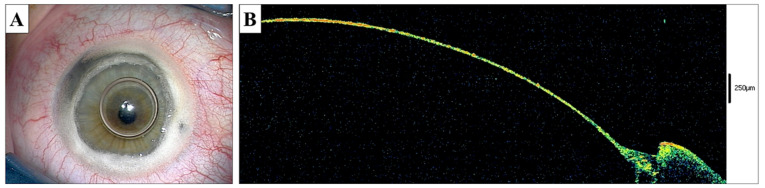
Big Bubble DALK procedure. (**A**)—The recipient’s bed prepared after intrastromal air injection and anterior corneal layer excision, the smooth Descemet’s surface visible over the air bubble in the anterior chamber. (**B**)—Intraoperative anterior OCT scan of the recipient’s bed showing the remaining tissue at approximately 20 µm of thickness.

Subsequently, the stromal layers were cut over the blunt spatula and inserted into the deep stroma. The stromal layers were removed with blunt-tipped microscissors. The bare Descemet’s membrane was visualized both with a surgical microscope as well as using intraoperative optical coherence tomography (OCT; iVue, Optovue Fremont, CA, US) (Figure 1A,B). The donor cornea was dissected before surgery using microkeratome (Moria Inc, Doylestown, PA, US), trephined to a size of 7.5 mm (Hessburg–Barron, Katena Products, Denville, NJ, US), and sutured into the recipient bed using double, continuous 10.0 nylon sutures.

In the PK group, the recipient cornea was trephined using a 7.25 mm Hessburg–Barron vacuum trephine (Barron Precision Instruments, Grand Blanc, MI, US). The vacuum trephine 7.5 mm (Hessburg–Barron, Katena Products, Denville, NJ, US) was used to trephine the donor cornea, which was sutured into the recipient bed using double, continuous 10.0 nylon sutures.

### 2.3. The Follow-Up Management

The postoperative management after both the DALK and PK procedures included topical 0.5% levofloxacin five times daily (Oftaquix, Santen, Finland), preservative-free 0.1% dexamethasone five times daily (Dexafree, Thea, France), and artificial tears eye drops (Tears Naturale II, Alcon, Fort Worth, TX, US). In the PK group, the patients additionally received daily 16 mg of systemic dexamethasone (Dexaven 4 mg/mL, Valeant, Canada) for a one week period. The topical antibiotic was discontinued one month after surgery, while topical dexamethasone was used over the following 6 months after DALK and 12 months after the PK procedure. Six months after the DALK surgery, the topical dexamethasone was replaced with a topical 0.5% loteprednol ophthalmic solution (Lotemax, Bausch&Lomb, Rochester, NY, USA) twice daily for the next 6 months. 

During the follow-up time, we evaluated the following parameters: best corrected visual acuity (BCVA, Snellen charts), refractive astigmatism (autorefractometer, Nidek AR-330A/10A, Nidek CO Ltd., 34-14 Maehama, Hiroishi-cho, Gamagori, Aichi 443-0038 Japan), intraocular pressure (Goldmann Applanation Tonometry), central corneal thickness (pachymetric map, Visante OCT, Carl Zeiss Meditec, Inc, Dublin, CA, USA), and endothelial cell count measured using a specular microscope (Topcon SP 3000, Topcon, Japan). Three specular photographs were taken, and each image was analyzed by selecting 50 endothelial cells. In addition to the quantitative parameters, we also evaluated the frequency of adverse events.

### 2.4. Statistical Analysis

SPSS 17.0 (SPSS Inc, Chicago, IL, USA) was used to analyze the data. All comparisons were made at the statistical significance level *p* < 0.05. The first stage of the statistical analysis was to evaluate the normality of the data distribution using the Shapiro–Wilk test (*p* > 0.05). According to these test results, we could use the parametric methods. Second, with the use of the ANOVA variance assay analysis, the differences shown were statistically significant, and next the post-hoc Tukey’s test was also conducted (*p* < 0.05). In order to analyze the differences between the two groups, Student’s *t*-test was performed (*p* < 0.05). The data are shown as the mean ± standard deviation (SD).

For calculating the correlation between CCT and BCVA, Pearson’s correlation coefficient was used (*p* < 0.05).

## 3. Results

### 3.1. Patients’ Characteristics

The mean age in the DALK group was not significantly different from the mean age in the PK group (28.6 ± 5.9 years and 28.4 ± 8.6; *p* = 0.76, Student’s *t*-test). The presence of systemic diseases, as well as the frequency of the familial occurrence of keratoconus were comparable (Table 2).

### 3.2. Visual Outcomes and Endothelial Cell Loss

The preoperative BCVA and logMAR in the DALK and PK groups were comparable. During the postoperative follow up, we observed significant and progressive improvement in the patients’ visual acuity (*p* < 0.005 and *p* < 0.0001, ANOVA analysis; 6 and 12 months after surgery, respectively). At the end-point of the follow-up (12 months), the BCVA and logMAR achieved in both groups were similar (*p* = 0.42, Student’s *t*-test) (Table 3).

Postoperative astigmatism measurements showed that the DALK procedure was burdened with significantly higher astigmatism (however, more regular) both 6 and 12 months after surgery when compared to patients that underwent PK surgery (*p* < 0.002, *p* < 0.001, Student’s *t*-test, respectively). To assess the regularity of astigmatism, we used routine criteria and methods, such as corneal topography of the anterior and posterior surfaces. The regularity of astigmatism affects the uncorrected visual acuity. The corrected focus can be similar, correcting astigmatism with, for example, hard contact lenses.

**Figure 2:**
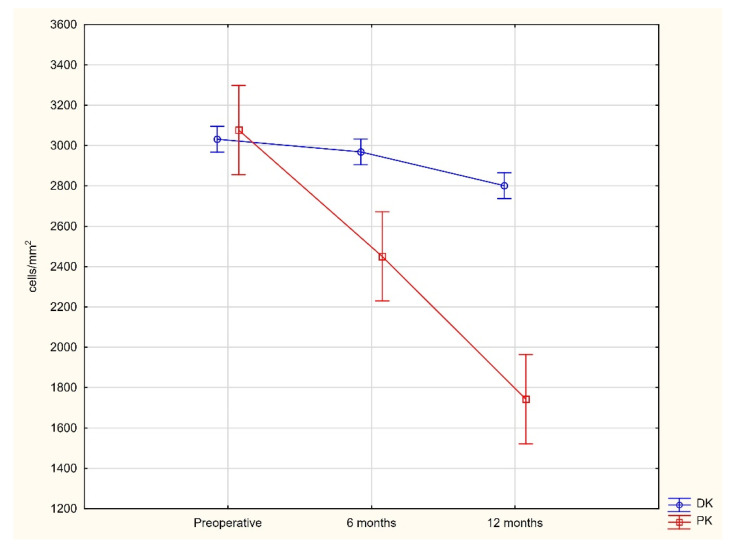
Changes in the number of endothelial cells in DK and PK patients during 12 months of observation (*p* < 0.05).

Although the initial mean endothelial cell density in the DALK group, estimated at the level of 3035.56 ± 254.9 cells/mm^2^, was slightly lower than in the PK group (3091.8 ± 263.86 cells/mm^2^), the postoperative endothelial cell loss (ECL) was significantly accelerated in patients from the PK group. Six months after surgery, the endothelial cell loss was significantly lower after the DALK procedure (3.78 ± 2.1%) when compared to PK (20.56 ± 10.11%; *p* < 0.0001, Student’s *t*-test). The ECL 12 months after surgery was still significantly lower after the DALK approach (7.49 ± 2.93%) when compared to PK (43.21 ± 13.04%; *p* < 0.0001, Student’s *t*-test) (Table 3; Figure 2).

### 3.3. Central Corneal Thickness Outcomes

Before surgery, the mean CCT was significantly lower in the PK group compared with in the DALK group (*p* < 0.0001, Student’s *t*-test) and remained significantly lower 6 and 12 months postoperatively (*p* < 0.0001, Student’s *t*-test). The preoperative BCVA and logMAR were not correlated with the CCT in both the DALK and PK groups (Pearson correlation coefficient r = 0.0896, *p* = 0.5 and *r*= −0.0919, *p* = 0.6, respectively). The postoperative increase in the CCT was 32.5 µm on average in DALK patients, while the increase in the PK group was approximately five-fold higher (*p* < 0.0001, Student’s *t*-test). Although the initial BCVA and logMAR did not depend on the CCT, 6 months after surgery, the visual acuity was positively correlated with the CCT in the DALK group but not in the PK group (*p* < 0.01 and *p* > 0.1, Pearson correlation, respectively).

### 3.4. Intraoperative and Postoperative Adverse Events

**Figure 3:**
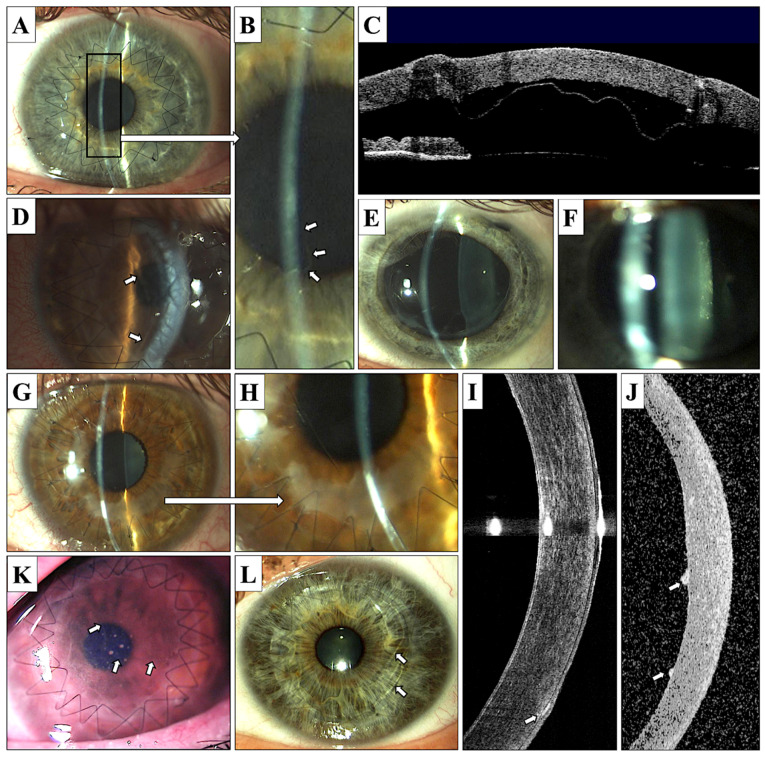
Postoperative complications of DALK and PK surgery. (**A**–**D**)—DALK, Descemet’s membrane detachment; (**A**)—localized, mild detachment (magnification presented on photograph B, arrows), (**C**)—severe Descemet’s membrane detachment visualized in anterior OCT (Visante); (**D**)—severe Descemet’s membrane detachment causing corneal graft edema (arrows). (**E**)—PK, Urrets–Zavalia syndrome; (**F**)—PK, posterior subcapsular cataract formation; (**G**–**I**)—DALK, subepithelial opacifications (**G**,**H**)—opacifications visible in area of sutures, mostly in the lower cornea; (**I**)—anterior OCT Visante, subepithelial opacifications visible in lower cornea, arrow); (**J**)—PK, anterior OCT Visante, endothelial sediments associated with endothelial graft disease; (**K**)—PK, endothelial graft disease with visible Khodadoust line (arrows) and endothelial sediments; and (**L**)—PK, Descemet’s membrane folds (arrows).

Intraoperative perforation of Descemet’s membrane occurred in eight (8%) of the eyes during the DALK procedure, and five of these cases (5%) required conversion to PK surgery. Early postoperative complications after DALK were represented by Descemet’s membrane detachment with double anterior chamber formation in six (12%) eyes (commonly diagnosed using anterior segment OCT) and Descemet’s membrane folds (Figure 3A–D,L).

Urrets–Zavalia syndrome occurred in one eye after DALK and in one after PK (Figure 3E). Urrets–Zavalia syndrome is a necrosis of the iris caused by the collapse of the blood vessels in the iris during mydriasis (dilated pupil) intraoperatively. Additionally, it is a coexisting disease with cave and cataracts. The patient has a permanently dilated pupil. Therefore, we do not dilate the pupil before the treatment of the cones. Secondary cataracts developed in two DALK patients (4%) and five PK patients (12.5%; Figure 3F). 

An episode of graft disease of the endothelial type occurred in five (12.5%) eyes after PK surgery and was not recorded after the DALK procedure; however, after lamellar keratoplasty, we observed the development of subepithelial opacities that appeared with relatively high frequency (in 26% of eyes) (Figure 3G–K). All patients with graft disease were treated with the administration of intense topical steroids, and no graft failure occurred. During the 12 months of follow-up, an elevated intraocular pressure (IOP), defined as values greater than 21 mmHg, appeared in four (8%) eyes after DALK and in 21 (52.5%) eyes after PK. Multiple complications appeared in one patient after DALK surgery and in four patients after the PK procedure. The postoperative adverse events are listed in Table 4.

**Figure 4:**
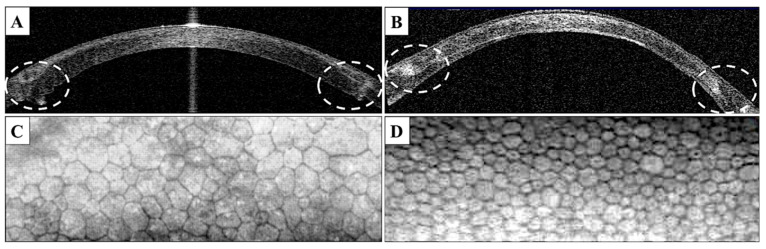
**(A**,**B**)—Anterior OCT Visante presenting the donor–host junction interaction after PK (**A**) and DALK (**B**), noticeable precise adjustment of the wound with no posterior folds visible in the DALK OCT scan, in contrast to a mismatch of the donor–host junction after PK. (**C**,**D**)—endothelial cell photographs from a specular microscope 12 months after PK (**C**) and DALK (**D**), visible different densities of endothelial cells, as well as polymorphism/polymegatism of the cells in the case of the PK patient.

In DALK transplant, the suturing results in no folding of the tissue margins, while after PK folds are clearly visible in OCT especially on the posterior transplant surface (Figure 4A,B).

## 4. Discussion

The results of the presented study favor deep anterior lamellar keratoplasty, which appeared to be a method with comparable visual outcomes, however, with higher refractive astigmatism, a lower final central corneal thickness, less frequent post-surgical intraocular pressure elevation episodes, and significantly lower endothelial cell loss as well as associated with a lower risk of endothelial graft rejection compared with penetrative keratoplasty.

An accurate patient qualification for corneal transplantation is one of the most important requirements for a satisfactory postoperative outcome. In the case of keratoconus, BCVA, the central corneal thickness and signs of Descemet’s membrane rupture should be the most considerable factors [6,7]. The borderline BCVA for the qualification of a keratoconus patient for keratoplasty is a matter of discussion. In their study, Reinchard et al. qualified patients for DALK procedure with BCVA ≤ 0.2, while other groups set the border at BCVA ≤ 0.3 [8,9]. 

We are convinced that a preoperative BCVA less than 0.3 (logMAR 0.52) is a value when the decision about surgery should be considered, and, for BCVA below 0.1 (logMAR 1.00), a decision about surgery is the most reasonable [10]. The central corneal thickness is an important risk factor for intraoperative complications for the big-bubble technique [11]. In our study, the minimal CCT value that allowed us to perform the big-bubble method was 350 µm. There are studies that describe performing DALK transplantation in patients with a minimal CCT of 250 µm; however, in our opinion, such a low CCT results in a higher risk of Descemet’s membrane perforation. 

The main intraoperative complication of the DALK procedure is associated with the necessity of transversion into a penetrative procedure [10,11]. The following important qualification criterion for DALK is the continuity of Descemet’s membrane. It is recommended that patients who have experienced hydrops episodes (i.e., severe corneal edema due to sudden Descemet’s membrane rupture) should be disqualified from the DALK surgery. 

Although there are some studies where the authors performed the DALK procedure after Descemet’s membrane perforation, in our opinion, preoperative Descemet’s membrane rupture should disqualify a patient from the lamellar procedure due to the higher risk of later endothelial graft rejection, as well as poorer visual outcomes associated with persistent deep stromal scarring [12]. On the other hand, even in the case of pre- or intraoperative Descemet’s membrane rupture, the fact that the postoperative endothelial cell loss after DALK procedure is significantly lower when compared to PK, this contraindication to DALK surgery should be always reviewed carefully, especially in young patients [13].

Visual outcome is one of the most important subjective criteria according to which a patient estimates the success of the surgery. In the presented study, there was a clear and significant improvement in the BCVA and logMAR in both groups (i.e., DALK and PK) 6 and 12 months after surgery; however, we did not find either of these two methods more beneficial according to the BCVA and logMAR outcomes. A constant increase of BCVA in the DALK group was previously shown to be noticeably dependent on the surgeon learning curve [14]. In our study, refractive astigmatism after DALK was significantly higher than after PK, both 6 and 12 month postoperatively, however, was observed to be more regular than after PK. 

Such conditions can be explained by the fact that, during DALK surgery in keratoconus, the perfect wound margin adjustment is almost impossible, because of the differences in the recipient bed depth and donor graft thickness. We found it interesting that, in anterior OCT scans, in the case of DALK there was much better interactions between the recipient and donor stroma on the cutting margin, which makes wound attachment and suturing more precise and results in more regular astigmatism, however, of higher value, due to the thickness mismatch. 

In DALK transplants, the suturing results in no folding of the tissue margins, while, after PK, folds are clearly visible in the OCT especially on the posterior transplant surface (Figure 3A,B). DALK is a new procedure, and PK is a procedure repeatedly performed by surgeons. In the case of DALK, the donor flap is implanted into the bed of the dissected cornea, while in the case of PK, it is sutured to the vertebra that has no bottom; therefore, wound adaptation may be better. The thickness of the donor’s cornea is not always the same as that of the recipient, and usually it is greater. The regularity of astigmatism is more important in assessing the UCVA—unadjusted visual acuity compared with the BCVA—best corrected visual acuity.

This phenomenon was noticed before by the Tan group and Borderie et al. [15,16]. However, there are surgeons who reported the achievement of similar astigmatism outcomes despite the surgical technique [17]. The central corneal thickness and thickness of the recipient bed remaining after corneal dissection can determine the visual outcomes after DALK. Ardjomand et al. [18] proved a correlation between the BCVA and CCT after DALK. In their study, the BCVA was significantly higher in the case of eyes with the remaining recipient bed smaller than 20 µm and tended to decrease when the bed was thicker than 80 µm [18]. 

In our study, the central corneal thickness was significantly lower in the DALK group compared with in the PK group after 6 and 12 months follow up and the absolute increase of the postoperative CCT was significantly higher after PK. This outcome is related to the fact that, in the case of PK, the initial CCT was much lower than in the DALK group. The explanation of the lower final CCT in the DALK group is related to the policy of the tissue bank, that one donor’s cornea is commonly used for two lamellar grafts (anterior DALK and posterior DSAEK), which is the cause of the reduced thickness of the DALK corneal button. 

In contrast to Ardjomand et al. [18], we did not find a clear correlation between the final BCVA and CCT both after DALK and PK; however, in the DALK group, the CCT influenced the BCVA during the healing process (i.e., there was a correlation between the CCT and BCVA observed at 6 months of follow-up). Since the thickness of the created recipient bed determines the BCVA more than the CCT itself, the precision of detaching Descemet’s membrane was supported in our study by the intraoperative anterior OCT that helped to unify the corneal layer separation outcomes and to control the thickness of the remaining recipient bed. Although the CCT in our DALK patients varied due to the thickness of donor cornea, the transplantation was performed based on a similar, comparable recipient bed.

Endothelial cell loss after penetrating keratoplasty is fully related to the primary quality of donor tissue (i.e., the donor age, initial endothelial cell density, and presence of endothelial pathology). The storage conditions of sclero-corneal buttons and the transplanting procedure can additionally accelerate the ECL. The great advantage of anterior lamellar keratoplasty is the possibility to preserve the recipient endothelium intact, which, in the case of keratoconus patients, is important, because their young age usually ensures a high density and a good condition of endothelial cells. 

The ECL estimation after surgery is reliable at least 6 months postoperatively, which is assumed to be the shortest time required to clear endothelial surface from the cells destroyed during surgical procedure. The ECL after DALK surgery in our patients equaled 4% after 6 months and accelerated up to 7% after 12 months postoperatively, while the physiological ECL was estimated at the level of approximately 1% per annum. The ECL outcomes after DALK surgery vary in the literature. 

Cheng et al. [13] presented 19% of the ECL after 12 months follow up; however, their study referred to patients after intrasurgical Descemet’s membrane perforation [13]. In contrast to DALK patients, PK patients developed ECL up to 43% after 12 months follow up. If we consider the initial EC density of 3091.8 ± 263.86 cells/mm^2^, the final EC density was approximately at the level of 1500 cells/mm2, creating a high risk of corneal graft decompensation (Figure 2 and Figure 3C,D).

The postoperative adverse events in our study were divided into early (up to 3 months) and late (after 3 months). The most common early complication that appeared in presented study was postoperative Descemet’s membrane detachment. The management of this adverse event, in the case of no spontaneous re-attachment is based on intracameral air or C3F8 gas injection; however, the last one is associated with a higher risk of sustained IOP elevation [19]. In our DALK patients, in four eyes, Descemet’s membrane attached spontaneously, and two patients required intracameral air injection; however, there were also patients that developed Descemet’s membrane folds without visible detachment. 

Unresponsive, dilated pupil, called Urrets–Zavalia syndrome was another reported adverse event [20]. Originally, this syndrome was described in keratoconus patients undergoing penetrating keratoplasty; however, lately Maurinio et al. observed it also in DALK patients [21]. Urrets–Zavalia syndrome after DALK surgery was linked with intrasurgical Descemet’s membrane perforation or postoperative Descemet’s membrane detachment [22,23]. Our observations are similar, since Urrets–Zavalia syndrome developed in one patient after PK and one patient after DALK surgery complicated with intrasurgical Descemet’s membrane perforation.

In the presented study, the most common late complication was Descemet’s membrane folds, with the intensity ranging from a single to numerous folds involving whole graft area. Similarly, as reported by other authors, the presence of these folds did not affect the visual outcome after keratoplasty [24]. The most serious adverse event, potentially threatening the graft survival, was the graft disease. In our study, PK-associated endothelial graft disease was recorded with a frequency of 12.5%. 

Despite the lack of cases of endothelial rejection after lamellar keratoplasty, we observed the development of subepithelial opacifications in 26% of DALK patients. Due to early diagnosis and intense treatment, there were no graft failures due to graft rejection in our study. The frequency of graft disease in keratoconus patients varies in the literature. According to Trimarchi et al., graft rejection occurs in 4% of cases after PK, and the risk of graft reject is not increased after DALK [25]. On the other hand, Watson et al. reported that, in their study, the risk of endothelial graft rejection after PK was 44% and after DALK 7.6% [26,27].

The other serious complication, associated with a high risk of permanent vision impairment is an increased intraocular pressure and secondary glaucoma. In our study, there was a significant difference in the frequency of IOP elevation, which was recorded in 8% of eyes after DALK and 52.5% of eyes after PK surgery. In the DALK group, three out of four cases showed good IOP control on topical medications (50 μg/mL latanoprost, Xalatan, Pfizer, New York, NY, USA), and one case required filtration surgery (trabeculectomy). In the case of the PK group, 30% had hypertension, including three patients requiring trabeculectomy. Others had well-controlled IOP on drugs (50 μg/mL latanoprost, Xalatan, Pfizer, New York, NY, USA). 

There was a huge difference here, probably related to the need for longer use of steroid drops. The significant difference between IOP after DALK and PK can be explained by the procedure techniques. During DALK, the anterior chamber is not entered by the surgeon, which reduces the risk of later synechiae formation and irido-corneal angle pathology, thereby, reducing the risk of aqueous humor outflow disturbance and secondary glaucoma development [28]. Furthermore, during DALK surgery, the donor tissue is not exposed to the anterior chamber, which allows for reduced topical corticosteroid use and prevents the development of steroid-induced ocular hypertension and secondary glaucoma [29].

## 5. Conclusions

In the current study, through comparing the outcomes after DALK and PK surgery in keratoconus patients, we showed that both procedures had similar visual acuity outcomes; however, the safety profile (including endothelial cell loss, risk of graft disease, intraocular pressure elevation, and the need for systemic treatment) favored the DALK procedure over PK. The DALK transplantation should be considered as an alternative and less invasive method compared with the PK method regarding surgical approaches in keratoconus treatment.

## Figures and Tables

**Table 1:** Inclusion criteria for the deep anterior lamellar keratoplasty and penetrating keratoplasty groups.

	Inclusion Criteria	Exclusion Criteria
DALK	BCVA < 0.3 logMAR < 0.52 Amsler’s grade keratoconus – III, IV Central corneal thickness > 350µm Endothelial cell density > 2000 cells/mm^2^ No signs of Descemet’s membrane rupture (no hydrops history, no corneal opacities)	Patients with other ophthalmic diseases that may affect postoperative outcomes, such as: amblyopic eye, cataract, glaucoma and retinal disorders Noncompliance patients
PK	BCVA < 0.3 logMAR < 0.52 Amsler’s grade keratoconus – III, IV Signs of Descemet’s membrane rupture (history of hydrops, corneal opacities)	Patients with other ophthalmic diseases that may affect postoperative outcomes such as: amblyopic eye, cataract, glaucoma and retinal disorders Noncompliance patients

DALK, Deep Anterior Lamellar Keratoplasty; PK, Penetrating Keratoplasty; BCVA, Best Corrected Visual Acuity.

**Table 2:** Patient characteristics.

	Surgical Approach	
	DALK	PK
Age (years)	28.6 ± 5.9	28.4 ± 8.6
Gender	♀:♂ 22:28	♀:♂ 16:24
Heredity (%)	Siblings (3) Parents (2)	Siblings (2) Parents (1)
Allergy (%)	General (32) - pollen (26) - drugs (3) - other (3)	General (29) - pollen (25) - drugs (3) - other (1)
Contact lenses intolerance (%)	11	9
Systemic diseases (%)	Atopic dermatitis (10) Asthma (9) Mitral valve prolapses (11) Hypothyroidism (2)	Atopic dermatitis (12) Asthma (4) Mitral valve prolapses (7) Arthritis (2)

**Table 3:** Preoperative visual acuity and postoperative visual outcome and endothelial cell loss in the DALK and PK groups.

		DALK (mean ± SD) *n* = 50	PK (mean ± SD) *n* = 40	P
BCVA	Preoperative	0.039 ± 0.037	0.038 ± 0.056	0.24
	6 months	0.49 ± 0.17	0.48 ± 0.17	0.48
	12 months	0.54 ± 0.17	0.52 ±0.14	0.41
logMAR	Preoperative	1.41 ± 1.43	1.42 ± 1.25	0.24
	6 months	0.31 ± 0.77	0.32 ± 0.77	0.48
	12 months	0.27 ± 0.77	0.29 ± 0.85	0.42
Refractive astigmatism (D)	6 months	2.50 ± 1.41	2.01 ± 0.94	0.00196
	12 months	2.48 ± 0.859	1.89 ± 0.86	0.00096
Endothelial cell loss cells/mm ^2^ (%)	Preoperative	3035.56 ± 254.92 (100)	3091.80 ± 263.86 (100)	0.98
	6 months	2920.82 ± 245.28 (3.78 ± 2.10)	2456.13 ± 209.61 (20.56 ± 10.11)	<0.0001
	12 months	2808.20 ± 133.49 (7.49 ± 2.93)	1755.83 ± 149.85 (43.21 ± 13.04)	<0.0001
CCT (µm)	Preoperative	(419.64 ± 31.34)	(369.60 ± 61.0)	<0.0001
	6 months	452.10 ± 89.14	528.87 ± 69.90	<0.0001
Astigmatism value	6 months	2.74 ± 1.42	2.02 ± 0.94	<0.002
	12 months	2.49 ± 0.86	1.89 ± 0.86	<0.001

DALK, Deep Anterior Lamellar Keratoplasty; PK, Penetrating Keratoplasty; and CCT, Central Corneal Thickness.

**Table 4:** Postoperative adverse events in patients undergoing DALK and PK corneal grafting.

	DALK (*n* = 50) N (%)	PK (*n* = 40) N (%)
Eyes with multiple complications	1 (2)	4 (10)
Subepithelial opacifications	13 (26)	0
Endothelial graft disease	0	5 (12.5)
Double anterior chamber	6 (12)	0
Descemet’s membrane folds	11 (22)	0
IOP elevation	4 (8)	21 (52.5)
Urrets–Zavalia syndrome	1 (2)	1 (2.5)
Secondary cataract	2 (4)	5 (12.5)

DALK, Deep Anterior Lamellar Keratoplasty; and PK, Penetrating Keratoplasty.

## Data Availability

The data used to support the findings of this study are included in the article. The data will not be shared due to third-party rights and commercial confidentiality.
